# The Role of Public Health in COVID-19 Emergency Response Efforts From a Rural Health Perspective

**DOI:** 10.5888/pcd17.200256

**Published:** 2020-07-23

**Authors:** Sandra C. Melvin, Corey Wiggins, Nakeitra Burse, Erica Thompson, Mauda Monger

**Affiliations:** 1 Institute for the Advancement of Minority Health, Ridgeland, Mississippi; 2 Mississippi State Conference of the NAACP, Jackson, Mississippi; 3 Six Dimensions, LLC, Ridgeland, Mississippi; 4 Magnolia Medical Foundation, Jackson, Mississippi; 5 MLM Center for Health Education and Equity Consulting Services, LLC, Jackson, Mississippi

## Abstract

As the country responds to coronavirus disease 2019 (COVID-19), the role of public health in ensuring the delivery of equitable health care in rural communities has not been fully appreciated. The impact of such crises is exacerbated in rural racial/ethnic minority communities. Various elements contribute to the problems identified in rural areas, including a declining population; economic stagnation; shortages of physicians and other health care providers; a disproportionate number of older, poor, and underinsured residents; and high rates of chronic illness. This commentary describes the challenges faced by rural communities in addressing COVID-19, with a focus on the issues faced by southeastern US states. The commentary will also address how the COVID-19 Community Vulnerability Index may be used as a tool to identify communities at heightened risk for COVID-19 on the basis of 6 clearly defined indicators.

**Summary**

**What is already known on this topic?**

Coronavirus disease 2019 (COVID-19) is a serious global pandemic. Rural minority communities are particularly at risk because of a weakened health care infrastructure, health care provider shortages, and lower socioeconomic status.

**What is added by this report?**

This report describes challenges faced by rural communities affected by the COVID-19 pandemic and provides recommendations to address those challenges.

**What are the implications for public health practice?**

The COVID-19 Community Vulnerability Index is a tool that can help identify communities most at risk for COVID-19 based on indicators such as socioeconomic status and health care system factors.

## Introduction

Rural communities are heterogeneous. In 2010, 19.3% of the US population resided in rural areas, compared with 54.4% in 1910, with the highest concentration being in the southeastern United States. The southeastern region includes Alabama, Arkansas, Florida, Georgia, Louisiana, Mississippi, North Carolina, South Carolina, and Texas, and racial and ethnic minorities make up 19% of the entire rural population (1). Socioeconomic characteristics influence the risk of infection with severe acute respiratory syndrome coronavirus 2 (SARS-CoV-2). For example, in Mississippi, approximately 20% of the population lives in poverty (2). In 2019, Mississippi, Louisiana, Arkansas, and Alabama were ranked as the country’s least healthy states (2). This statistic is important, because the less healthy the population, the more likely the epidemic is to have fatal consequences. In addition, the weaker the health system, the harder it is to contain the virus.

Most of the states that make up the southeastern United States are rural (Table 1). Rural communities face a unique set of challenges in the face of the coronavirus disease 2019 (COVID-19) pandemic. They are often areas already affected by high levels of poverty, lower levels of access to quality health care, lower levels of health literacy, and social stigma. Many elements contribute to these problems, including a declining population; economic stagnation; shortages of physicians and other health care professionals; a disproportionate number of older, poor, and underinsured residents; and high rates of chronic illness. This commentary will describe the challenges and issues faced by rural communities in addressing the COVID-19 pandemic. It will also show how the COVID-19 Community Vulnerability Index (CCVI) (4) may be used as a tool to identify communities at highest risk for COVID-19 on the basis of 6 clearly defined indicators (Table 2).

**Table 1:** Percentage of Urban and Rural Populations in 9 Southeastern US States, 2010^a^

State	Total Population	Urban	Rural	% Rural
Alabama	4,779,736	2,821,804	1,957,932	41
Arkansas	2,915,918	1,637,589	1,278,329	44
Florida	18,801,310	17,139,844	1,661,466	9
Georgia	9,687,653	7,272,151	2,415,502	25
Louisiana	4,533,372	3,317,805	1,215,567	28
Mississippi	2,967,297	1,464,224	1,503,073	27
North Carolina	9,535,483	6,301,756	3,233,727	51
South Carolina	4,625,364	3,067,809	1,557,555	34
Texas	25,145,561	21,298,039	3,847,522	15

a Source: American Community Survey (3).

**Table 2:** COVID-19 Community Vulnerability Index Applied to 9 Southeastern US States^a^

States	Theme 1: SES	Theme 2: Household Composition and Disability	Theme 3: Minority Status and Language	Theme 4: Housing Type and Transportation	Theme 5: Epidemiological Factors	Theme 6: Health Care System Factors	CCVI
Alabama	0.92	0.86	0.42	0.28	0.88	0.96	1
Arkansas	0.9	0.98	0.36	0.82	0.84	0.82	0.96
Florida	0.68	0.42	0.88	0.52	0.46	0.9	0.9
Georgia	0.84	0.56	0.78	0.36	0.44	0.92	0.86
Louisiana	0.94	0.88	0.52	0.66	0.98	0.38	0.88
Mississippi	1	1	0.46	0.74	0.92	0.5	0.92
North Carolina	0.8	0.66	0.68	0.62	0.48	1	0.98
South Carolina	0.86	0.84	0.5	0.5	0.54	0.54	0.64
Texas	0.72	0.44	0.96	0.46	0.38	0.7	0.8

Abbreviations: CCVI, COVID-19 Community Vulnerability Index; SES, socioeconomic status.

a CCVI scores range from 0 to 1; higher scores indicate greater vulnerability. Source: Surgo Foundation (4).

## Challenges for Rural Communities

As the COVID-19 outbreak continues to place a burden on hospitals throughout the United States, concern is growing that many hospitals, in particular rural hospitals, may not have the financial reserves to remain fiscally viable. Most rural hospitals operate on tight budgets, and they rely on high-profit services, such as elective surgery, to keep them in business. For many rural hospitals, canceling these profitable services to cope with the COVID-19 pandemic may result in financial catastrophe (5).

The closure of rural health care facilities or the discontinuation of services can negatively affect access to health care in a rural community. People in rural areas who get sick with COVID-19 have fewer hospitals to treat them. Compared with urban hospitals, rural hospitals are smaller, have a higher proportion of primary care physicians and a lower proportion of board-certified physicians on their medical staffs, have fewer intensive care beds, and are less likely to have contracts with health maintenance organizations and preferred provider organizations.

People living in rural areas are at increased risk of COVID-19, because they are less likely to be employed and more likely have low incomes than people living in other areas. They also face significant barriers to accessing care, including provider shortages, recent closures of rural hospitals, and long travel distances to providers. Local rural health care systems are fragile; when one facility closes or a provider leaves, it can affect care and access to care throughout the community. Furthermore, when a hospital closes, access to nonhospital care can also decline, because many specialists cluster around hospitals. Rural hospitals face severe financial challenges, and they are also more likely than urban hospitals to close. For example, 15 of 21 hospitals that closed in the United States in 2016 were in rural communities, and since 2010, nearly 90 rural hospitals in the United States have closed (6). Another financial challenge to rural hospitals is shrinking populations, which means fewer patients to fill beds. Although populations in urban counties have increased since 2000, populations in half of rural counties in the United States have decreased, which has caused a reduction in revenue for rural hospitals. Most recent hospital closings have been in states that opted not to expand Medicaid under the Affordable Care Act, which means that a significant portion of their health care costs remain uncompensated, thus creating a financial burden for these states (7).

Given the unique challenges for rural communities — exacerbated by a weakening rural health care infrastructure, health care provider shortages, and closure of rural hospitals — monitoring and control plans need to be developed to ensure that the magnitude of illness and death in those communities are assessed. Specifically, solutions need to be developed that account for the rural nature of these communities as well as the social determinants of health that influence health care outcomes.

## COVID-19 Community Vulnerability Index

Community-level social disadvantage and vulnerability to disasters can influence the incidence of COVID-19 and its adverse outcomes in several ways. For example, lower socioeconomic status (SES) is associated with poor health care access, which may increase risk for adverse health outcomes. Labor inequalities, lack of workplace protections, and household overcrowding may decrease the ability to adhere to social-distancing guidelines. Additionally, racial/ethnic minorities and immigrants are less likely to have access to appropriate and timely health care. Evidence suggests that these inequalities contributed to disease spread and severity during the H1N1 influenza pandemic (8–11).

The CCVI, developed by the Surgo Foundation (4), can be used to identify which communities may need the most support during a pandemic or similar public health emergency. CCVI scores range in value from 0 to 1, with higher scores indicating greater vulnerability. A given geographic unit — for example, a census tract or county — is ranked relative to all similar units across the country on the basis of 6 themes: 1) SES, 2) household composition and disability, 3) minority status and language, 4) housing type and transportation, 5) epidemiologic factors, and 6) health care system factors. The score generated can then be used to designate a level of vulnerability. Each designation corresponds to a quintile of that geographic unit type in the United States. For example, a county score of 0 to 0.20 would correspond to very low vulnerability compared with all other US counties, a score of 0.21 to 0.40 would correspond to low vulnerability, and so on through the last category of very high vulnerability and a score of 0.81 to 1.

The CCVI is not designed to predict which individuals will become infected with SARS-CoV-2. However, it can provide information about the anticipated negative impact at the community level. This information can help decision makers target resources where they are most needed. The index could be useful in developing a community risk profile for SARS-CoV-2 infection that can be used to target and tailor control efforts. Data from the CCVI demonstrate that each of the 9 southeastern states has a CCVI score that indicates very high vulnerability. Scores for each state also indicated very high vulnerability on each of the 6 indicators used to generate the CCVI (4,12–14). For example, Mississippi has a score of 1 for SES and household composition and disability and a score of 0.92 for epidemiologic factors. The overall CCVI score for Mississippi is 0.92. This score indicates that Mississippi is particularly vulnerable and prone to poorer COVID-19–related outcomes, especially in communities with lower SES and poor health status overall.

Since the outbreak of COVID-19, health care delivery has changed considerably. The United States has adapted its technology and policies to accommodate health care delivery at a distance. However, although telehealth use has increased during the pandemic, the regulatory changes that made this increase possible are not permanent. Moreover, the kinds of technologic advancements required for remote health care delivery can be challenging to implement in rural communities. The terrain can make it difficult, sometimes impossible, to install fiber or other infrastructure, and the biggest barrier to obtaining broadband internet service in certain areas of the country is low population density.

Furthermore, the cost of telemedicine for rural health clinics is an issue, because many rural patients receive either Medicare or Medicaid, and reimbursements from these government health care programs, as well as from private insurance companies, do not fully cover the costs of virtual medicine.

For rural communities in the Southeast, success at implementing these virtual systems has been fragmented. Unreliable access to at-home technology, broadband internet service, and cellular telephone reception have prevailed in some communities, while ever-present financial hurdles abound. The COVID-19 pandemic has exposed the limitations of these remote areas (15).

## Special Concerns for Rural Communities

Affordability of health care is a significant challenge for rural areas in the southeastern United States. However, several of the most rural states in the country opted not to expand Medicaid under the Affordable Care Act; 59% of uninsured rural people live in these states (16). Lack of insurance has implications for access to care, because people without health insurance may delay seeking care even if they have symptoms, for fear of incurring expenses that they cannot pay (16).

In addition to lacking good health insurance, many people living in southeastern and rural states face the barrier of distance (17). Geographic isolation and related challenges, including lack of transportation and extreme weather conditions, make it harder for people in rural communities than people in urban communities to travel for care, and services are typically farther away (18). For example, to get to Sunflower Medical Center in Ruleville, Mississippi, some patients travel as far as 45 miles to receive care (15).

The lack of infrastructure is not limited to roads and highways; in rural areas, health care infrastructure may also be extremely limited, health care resources scarce, and clinical providers few. Only 9% of the nation’s physicians and 16% of the nation’s registered nurses practice in rural areas. Dentists and pharmacists are also scarce in these areas (18).

## Implications for Public Health

Community health centers play an important role in rural and remote areas and form one of the largest systems of care available to rural populations. Today, community health centers serve 1 in 6 rural residents (19), so they have a critical role in the response strategy to COVID-19 in rural communities. Because health centers are in virtually every community in our country, they are in a unique position to respond to COVID-19. They can help increase access and availability of COVID-19 testing for the community.

However, despite ramping up testing and virtual visits, health centers are reporting steep declines in patient visits, and many staff members are unable to work because of COVID-19–related issues. These issues include having to juggle work obligations and parenting obligations as a result of school closings and not being able to find appropriate child care as a result of day care closings. Another challenge is the temporary closures of health centers as a result of the pandemic. Although health care centers received $1.98 billion in rapid response grants from the federal government, more financial support may be needed to sustain services (20). Health centers also have issues related to the availability of personal protective equipment and testing supplies. Staffing to assist with contact tracing for COVID-19–positive people is also necessary.

The CCVI is a valuable tool that can be used as part of a coordinated response to identify communities at greatest risk for COVID-19, so that resources can be deployed strategically to those areas. This tool, in coordination with targeted testing and contact tracing, can be effective in flattening the COVID-19 curve and ensuring that the most vulnerable communities have access to health care resources. Creating a complete profile of people at risk for SARS-CoV-2 infection is also important. A complete risk profile, including geographic hotspots, needs to be developed for the southeastern region to target and tailor control efforts.

Stakeholders that work with underserved populations should be included in the emergency response planning process and enlisted to help reach disadvantaged and marginalized communities. Information generated from the CCVI can be used to develop a coordinated, comprehensive approach to addressing the pandemic that is specific to rural communities in the South. These stakeholders should include hospitals, health care centers, insurance providers, policy makers, community-based organizations, and faith-based organizations. This coordination would be valuable in planning emergency response, identifying areas of greatest needs, developing culturally appropriate messaging, and disseminating information throughout the community.
